# Stability of a new cubic monoxide of Thorium under pressure

**DOI:** 10.1038/srep13740

**Published:** 2015-09-04

**Authors:** Weiwei Sun, Wei Luo, Rajeev Ahuja

**Affiliations:** 1 Department of Material Science and Engineering, KTH-Royal Institute of Technology, Stockholm SE-10044, Sweden; 2 Department of Physics and Astronomy, Uppsala University, Box 516, SE-75120 Uppsala, Sweden

## Abstract

Density functional theory has been applied to elucidate the stability of thorium monoxide (ThO). It is found out that the pressure can stabilize the rocksalt phase of ThO, and the transition pressure is estimated between 14 and 22 GPa. The stability of ThO can be attributed due to the gradually filling 5*f* orbitals at the expense of 7*s* and 6*d* electrons in Th metal. For ThO, the pressure induces stronger Th-O bond reflected by the newly established 6*d*-2*p* hybridization which is the dominant cause of its stability. The phonon dispersion curves of the rocksalt phase show the positive frequencies which indicates its dynamical stability. Our successful prediction of the stabilization of the metallic ThO has proposed a route to synthesize novel actinide monoxides.

Actinide compounds having many versatile properties, such as polymorphism, non-stoichiometry and intermolecular in nature have been becoming a crucial and challenging scientific topic from basic as applied point of view specially in nuclear industry^1^. Facing the challenge of consuming of Uranium (U) and the requirement for the coming Generation IV reactors, Th related materials have been focused on and attempted to be fabricated as the future nuclear fuel materials. One of the advantages of Th over U is the much higher abundance of Th in the earth crust. One can also expect that the less nuclear waste can be produced. The nuclear fuels serve the transmutation function by incorporating components from the promising fuel materials. In this scenario, Th has also been recognized as a powerful alternative for many years^2^. The thorium fuel cycle is attracting unique and positive interest from Russia, China, and India. Briefly, there are two main choices for the thorium fuels: once through cycle using (Th, U) oxide fuel; or a plutonium consumption cycle, namely thorium-assisted plutonium incineration^3^. It is well known that this heavily reduced MA (Minor Actinides e.g., Np, Am and Cm) production is one of the benefits of using thorium fuels because the MA contribute significantly to the burden of wastes in the long time scale^4^,^5^. It is also highlighted that the small-scale chemical reprocessing of irradiated thorium has shown a great advantage^6^. Therefore, the investigations of current and new actinide oxides play the crucial role in the nuclear fuel cycle in the coming years.

Titanium, zirconium, hafnium and cerium, which reside in the same *d*2 column all have their monoxides but thorium does not form the monoxide (ThO). Until now, only thorium dioxide being the only oxide has been extensively investigated both experimentally^7^,^8^ and theoretically^9^,^10^. So far, a few theoretical and experimental studies^11^,^12^ have paid attentions to the gas phase of ThO, and many attempts to synthesise solid ThO have failed. It was figured out that only partial oxygen concentration (ThO_1−*x*_) was achieved in study of by Ackermann *et al.*^13^ and at absolute zero temperature, the dissociation of ThO into Th and ThO_2_ is favored^14^,^15^,^16^. In this work, we evaluated the reaction enthalpy of the following chemical reaction:


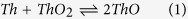


As it is mentioned above, the natural reaction is the dissociation of ThO. At ambient conditions, ThO can not be formed. Nowadays pressure is used routinely as an efficient tool to stabilize the novel materials at extreme conditions. So, we have computationally studied the reaction (1) under pressure. In fact, high pressure exerted on the system can vary the reaction enthalpy as well as lower the activation energy by overcoming energy barrier, which results from the induced structural changes of the reactants. Hereby, the pressure has been applied to the above reaction. It is known from literature that Th is stable in fcc structure up to 100 GPa^17^, whereas the fluorite (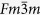) phase of ThO_2_ transforms to the orthorhombic *α*-PbCl_2_ phase at 40 GPa^18^. These studies imply that no phase transition occurred in the pressure range of 0 ~ 35 GPa. The main aim of the present paper is to study the reaction enthalpy of ThO as a function of pressure in order to evaluate the static stability of ThO. Lattice dynamical calculations are performed to check the dynamical stability of ThO. The transformations of orbital occupations of the reactants and product are analysed within the crystal field theory framework. Our results show that the external pressure plays a dominating role in stabilization ThO.

## Methods

Throughout this work, we have performed first principles calculations based on density functional theory (DFT). We have treated the 5*f* states as the valence electrons since the 5*f* electrons have been found to stabilize fcc phase of Thorium metal in our previous work^19^. All calculations are performed in the fully relativistic calculations in local density approximation (LDA)^20^, and Perdew-Burke-Ernzerhof^21^ exchange-correlation functional in the RSPt code^22^,^23^, which employs a full potential linear muffin-tin orbital method (FP-LMTO)^24^,^25^. We have considered a constant muffin-tin radius to make all results comparable, and the spin-orbit coupling is taken into account as well. PHON^26^ package has been used to examine the dynamical stability in the framework of small displacement method.

## Results and Discussion

### Chemical reaction under pressure, and the dynamical stability

**Figure 1:**
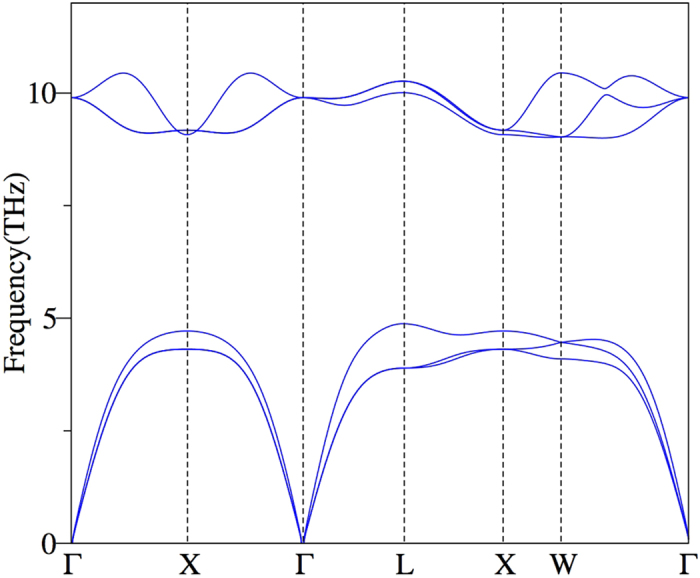
The phonon dispersion curves of ThO at 20 GPa.

The reaction enthalpy defined as 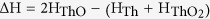 has been computed as a function of pressure up to 35 GPa. The reaction enthalpy is positive at ambient pressure which suggests that ThO is unstable. Now, when pressure increases and reaches around 14 GPa (LDA) and 22 GPa (PBE), the reaction enthalpy becomes negative which implies the stabilization of ThO. These two stabilization pressure with two different exchange correlation potentials can be used as lower and upper limits of stability range of ThO. Based on these results, we can confidently say that the transition pressure lies within this range. We can take an intermediate value of 20 GPa (LDA) as the prototype pressure to carry out the following analysis. To begin with, the dynamical stability of energetically stable ThO is tested in the full phonon calculations as described in Fig. 1. This figure shows that the phonon dispersion curves of ThO have no imaginary frequencies. In the phonon dispersion curve, the flatness along L-X-W also reminds that the chemical bonding in this path is rather isotropic (the XY plane). Another feature is the big frequency gaps between higher frequencies and acoustic modes that can be explained by the big mass ratio between Th and O. The longitudinal acoustic (LA) vibration at point L formed the frequency edge, the value of higher frequency is precisely double LA frequency. Therefore, ThO is stable in rocksalt structure.

**Figure 2:**
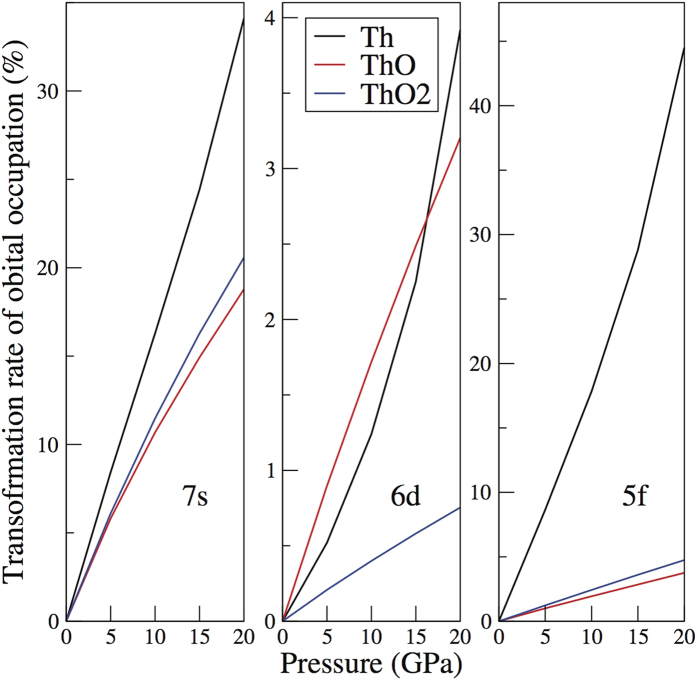
The transformation rate of the occupation number for 7*s* (left), 6*d* (middle), and 5*f* (right) orbitals in Th for Th, ThO and ThO_2_ as a function of pressure. The black curves represent Th, the red for ThO, and the green for ThO_2_.

The reaction can be further explained by the the behavior of each reactant and product under pressure. In fact, there are a few studies describing the behavior of Th^27^,^28^ under pressure. The 5*f* orbitals are filled at the expense of *d* electrons were reported therefore whereas the 7*s* orbital is not discussed specifically. In Fig. 2, we have shown the occupation number of 7*s*, 6*d* and 5*f* orbitals in Th, ThO_2_ and ThO, respectively. The plots reveal that the occupation number for 5*f* orbital increases as the occupations in 7*s* and 6*d* orbitals decreases as a function of pressure. Moreover, 7*s* electrons mainly contribute to the transformation compare to 6*d* electrons. In this regard, it can be linked to that the phase transition of fcc metallic Th (*spd* metal) to low symmetry bct metallic phase^27^. Stability of low symmetry bct phase indicates that *f* electrons are now dominant and participating in bonding. The results related to the transformation of 7*s* and 6*d* to 5*f* have also been reported in literature^29^. This kind of transformation is not very dominant in the case of ThO and ThO_2_. It is clear that the most inert phase is the highest oxidation state, i.e. ThO_2_ (no report of sesquioxide or higher oxidation states), which has the lowest transformation rate to 5*f* orbital. Naturally, the Th-O interaction alters the original behavior in Th (see further discussions). On the other hand, on the basis of the low occupation in 5*f* shell, the setting of two boundaries of LDA and PBE is reasonable and further beyond single-particle methods, like LDA + DMFT is not needed. In a nutshell, the distinctive transformations of 7*s*, 6*d* and 5*f* orbitals are correlated to the energies of Th, ThO and ThO_2_, which determine the enthalpy change.

### Electronic structure and the origin of the stabilization

**Figure 3:**
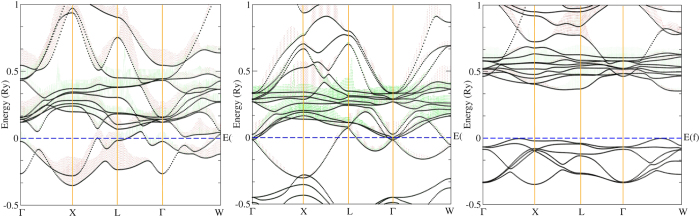
The band structures of Th, ThO and ThO_2_ at 20 GPa with the weighted 6*d* (red) and 5*f* (green) bands.

The band structures of Th, ThO and ThO_2_ at 20 GPa along Γ-X-L-Γ-W directions are shown in Fig. 3, where the 6*d* and 5*f* weighted bands are in green and red colors, respectively. The comparison of the 6*d* bands within these three substances shows that the oxidation on Th leads to a strong excitation to higher energy scale. If the 5*f* bands in the three substances are compared, it is clear that the oxidation can compress its band width step by step from Th, ThO and to ThO_2_. Increase in oxidation prompts us to look in to the crystal field effects associated with the ligands, namely the number of oxygen atoms. In the structure of ThO, each thorium atom is surrounded by six oxygen atoms while for ThO_2_, each thorium atom has eight coordinated oxygen atoms. Hence, we simply employ the crystal field splitting (CFS) that the *d*/*f* bands split by a stronger strength in a higher number of coordination number of ligands. Since the 5*f* bands are becoming narrower with the increase of oxygen atoms per Th atom, the CFS of 5*f* orbital is marginal in the contrast to that of 6*d* orbital. This can be explained by the rather small occupation number of 5*f* orbital, therefore 6*d* orbital is the core of the oxidation. In ThO_2_, we observe one more interesting finding is that the valence band maximum are located at two points along Γ-X and Γ-W direction and the conduction band minimum are located at X-L and Γ-W direction. It will be very interesting to investigate the absorption spectrum of ThO_2_ to see whether the higher oxidation compound, ThO_2_ having a large band gap is due to the stronger 6*d* CFS or not.

**Figure 4:**
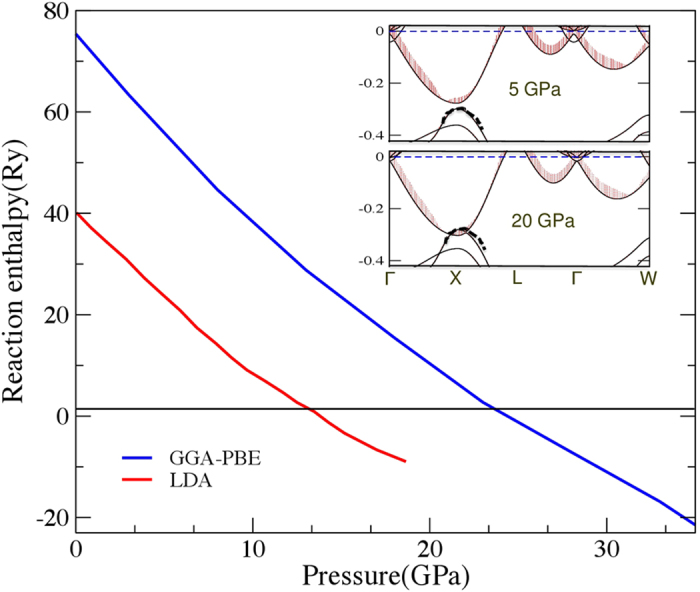
The reaction enthalpy obtained from LDA and GGA-PBE under pressure. The inner panel shows the band structures of ThO at 5 GPa (upper) and 20 GPa (lower). The weighted 6*d* bands are coloured in red, and the dashed dotts in black stand for O-2*p* bands.

To answer the above questions, the closer look at density of states is required, while so far, the mystery of stabilization ThO under pressure is still unveiled. In Fig. 4, the blurring spots are shown, and particularly the band shift in ThO under pressure is the core of the stabilization of ThO. Two different exchange-correlation (xc) functionals predict different transition pressures, and this can be used as the lower and upper limit of transition pressure. The inner panel presents the band shift of ThO under pressure, from which the origin of stabilizing ThO may be understood. At X point, the pressure forces the Th-6*d* and O-2*p* bands to mix together and this shift coincides with the 6*d*-2*p* mixing found in the stable phases of Thorium carbide and nitride^30^ at ambient conditions. So, we can conclude that hybridization of 6*d*-2*p* states in ThO serves as the dominant stabilizing factor.

**Figure 5:**
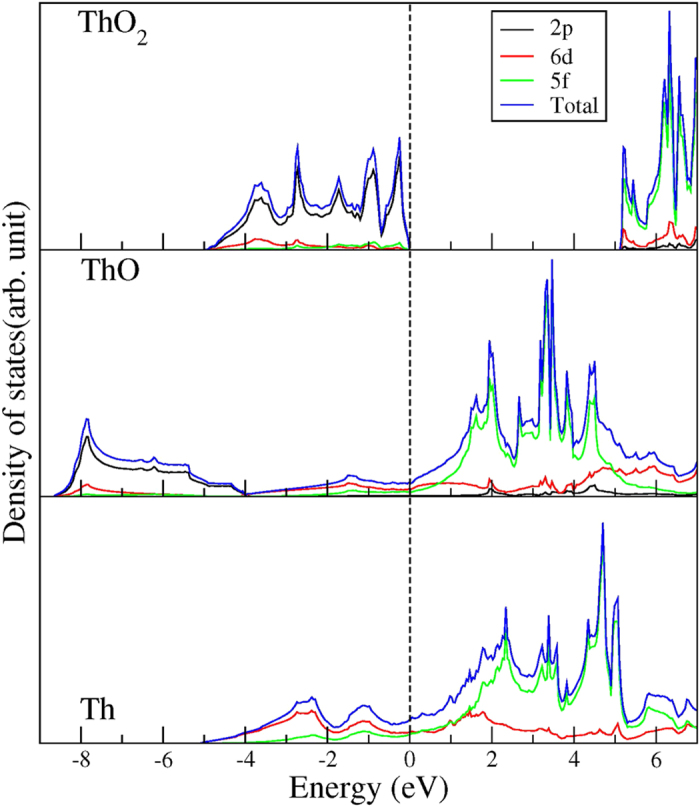
The total (blue) and projected density of states (PDOS) of 6*d* (red), 5*f* (green) and 2*p* (black) states in Th, ThO, ThO_2_.

The projected density of states (PDOS) can provide a profound description of the electronic structures of Th, ThO and ThO_2_ as shown in Fig. 5. In Th and ThO, it has been found that 6*d* states are rather delocalized, and the 6*d* along with 5*f* states primarily govern the DOS at E_*f*_. The 5*f* states mainly prevail at the conduction band with a tail entering in the valence band for ThO. A strong hybridisation between 6*d* and 5*f* states can be found in Th metal at −1 eV, which plays the most crucial role in the charge transfer which is reflected in Fig. 2. In ThO, the 6*d*-5*f* states show a peak at −1.8 eV and cut the E_*f*_ and extend to the conduction band. Once the oxidation of Th metal is initiated, the characteristics of this electronic structure altered. The introduction of oxygen to Th leads to the 6*d*-2*p* hybridisation at −8 eV, and the anti-bonding states at 2 eV and above. Under further oxidation from ThO to ThO_2_, the 6*d*-2*p* and 5*f*-2*p* hybridisation are pushed towards to the E_*f*_. This can be associated with the larger crystal field splitting between 6*d* and 2*p* interaction. Overall, the oxidation does impact on the electronic structure, and it turns Th metal into an insulator ThO_2_. The newly found ThO combines the characteristics of Th metal and ThO_2_.

## Conclusion

In summary, we have theoretically predicted the rocksalt like ThO to be stable in the pressure range between 14 GPa and 22 GPa. This virtual synthesise is performed based on the oxidation and reduction reaction between Th and ThO_2_. The change of energetics and dynamical stability from phonon dispersion analysis under pressure indicate the existence of ThO at the intermediate pressure value of 20 GPa. The main driver of the stability of ThO is found to be the emergence of 6*d*-2*p* hybridization. The 6*d* and 5*f* states behave differently from oxygen free to 2:1 oxidation states of Thorium. DOS reveals the metallic property of the stable ThO phase and the effects of pressure. The crystal field splitting makes ThO metallic. The metallic ThO shows greater thermal conductivity, which is one of the essential property for the future nuclear fuel materials. Our theoretical prediction of existence of Thorium monoxide has paved a new way to prepare such actinide monoxides that can be potentially used in the nuclear industry.

## Additional Information

**How to cite this article**: Sun, W. *et al.* Stability of a new cubic monoxide of Thorium under pressure. *Sci. Rep.* **5**, 13740; doi: 10.1038/srep13740 (2015).
